# CD4:CD8 Ratio and CD8 Count as Prognostic Markers for Mortality in Human Immunodeficiency Virus–Infected Patients on Antiretroviral Therapy: The Antiretroviral Therapy Cohort Collaboration (ART-CC)

**DOI:** 10.1093/cid/cix466

**Published:** 2017-07-11

**Authors:** Adam Trickey, Margaret T May, Philipp Schommers, Jan Tate, Suzanne M Ingle, Jodie L Guest, M John Gill, Robert Zangerle, Mike Saag, Peter Reiss, Antonella d’Arminio Monforte, Margaret Johnson, Viviane D Lima, Tim R Sterling, Matthias Cavassini, Linda Wittkop, Dominique Costagliola, Jonathan A C Sterne, for the Antiretroviral Therapy Cohort Collaboration (ART-CC), Andrew Boulle, Christoph Stephan, Jose M Miro, Matthias Cavassini, Geneviève Chêne, Dominique Costagliola, François Dabis, Antonella D’Arminio Monforte, Julia del Amo, Ard Van Sighem, Jorg Janne Vehreschild, John Gill, Jodie Guest, David Hans-Ulrich Haerry, Robert Hogg, Amy Justice, Leah Shepherd, Niels Obel, Heidi M Crane, Colette Smith, Peter Reiss, Michael Saag, Tim Sterling, Ramon Teira, Matthew Williams, Robert Zangerle, Jonathan Sterne, Margaret May, Suzanne Ingle, Adam Trickey

**Affiliations:** 1 School of Social and Community Medicine, University of Bristol, United Kingdom;; 2 Department I for Internal Medicine, University Hospital of Cologne, Germany;; 3 Yale University School of Medicine, West Haven, Connecticut;; 4 HIV Atlanta Veterans Affairs Cohort Study, Atlanta Veterans Affairs Medical Center, Decatur, Georgia;; 5 Division of Infectious Diseases, University of Calgary, Alberta, Canada;; 6 Innsbruck Medical University, Austria;; 7 Division of Infectious Disease, Department of Medicine, University of Alabama, Birmingham;; 8 Stichting HIV Monitoring, and Division of Infectious Diseases and Department of Global Health, Academic Medical Center, University of Amsterdam, and Amsterdam Institute for Global Health and Development, The Netherlands;; 9 Clinic of Infectious Diseases and Tropical Medicine, San Paolo Hospital, University of Milan, Italy;; 10 Department of HIV Medicine, Royal Free London NHS Foundation Trust, United Kingdom;; 11 British Columbia Centre for Excellence in HIV/AIDS, and Division of AIDS, Faculty of Medicine, University of British Columbia, Vancouver, Canada;; 12 Vanderbilt University School of Medicine, Nashville, Tennessee;; 13 Service of Infectious Diseases, Lausanne University Hospital and University of Lausanne, Switzerland; and; 14 INSERM, Unit of Epidemiology and Biostatistics, Bordeaux, and; 15 Sorbonne Universités, INSERM, UPMC Université Paris 06, Institut Pierre Louis d’épidémiologie et de Santé Publique (IPLESP UMRS 1136), Paris, France

**Keywords:** CD8 count, CD4:CD8 ratio, mortality, HIV, antiretroviral therapy

## Abstract

**Background:**

We investigated whether CD4:CD8 ratio and CD8 count were prognostic for all-cause, AIDS, and non-AIDS mortality in virologically suppressed patients with high CD4 count.

**Methods:**

We used data from 13 European and North American cohorts of human immunodeficiency virus–infected, antiretroviral therapy (ART)–naive adults who started ART during 1996–2010, who were followed from the date they had CD4 count ≥350 cells/μL and were virologically suppressed (baseline). We used stratified Cox models to estimate unadjusted and adjusted (for sex, people who inject drugs, ART initiation year, and baseline age, CD4 count, AIDS, duration of ART) all-cause and cause-specific mortality hazard ratios for tertiles of CD4:CD8 ratio (0–0.40, 0.41–0.64 [reference], >0.64) and CD8 count (0–760, 761–1138 [reference], >1138 cells/μL) and examined the shape of associations using cubic splines.

**Results:**

During 276526 person-years, 1834 of 49865 patients died (249 AIDS-related; 1076 non-AIDS-defining; 509 unknown/unclassifiable deaths). There was little evidence that CD4:CD8 ratio was prognostic for all-cause mortality after adjustment for other factors: the adjusted hazard ratio (aHR) for lower vs middle tertile was 1.11 (95% confidence interval [CI], 1.00–1.25). The association of CD8 count with all-cause mortality was U-shaped: aHR for higher vs middle tertile was 1.13 (95% CI, 1.01–1.26). AIDS-related mortality declined with increasing CD4:CD8 ratio and decreasing CD8 count. There was little evidence that CD4:CD8 ratio or CD8 count was prognostic for non-AIDS mortality.

**Conclusions:**

In this large cohort collaboration, the magnitude of adjusted associations of CD4:CD8 ratio or CD8 count with mortality was too small for them to be useful as independent prognostic markers in virally suppressed patients on ART.

## Summary

Associations of CD4:CD8 ratio or CD8 count with all-cause and cause-specific mortality were too small for them to be useful as independent prognostic markers in addition to CD4 count in virally suppressed patients on antiretroviral therapy with high CD4 count.

CD4^+^ T-lymphocyte (CD4) counts and human immunodeficiency virus type 1 (HIV-1) RNA measurements (viral load [VL]) are the most important prognostic markers for disease progression and recovery after starting combination antiretroviral therapy (ART) in people living with HIV (PLWH) [1–4]; most AIDS-related deaths occur in patients with CD4 count <350 cells/μL [5]. However, PLWH on ART with CD4 count >350 cells/μL and suppressed VL experience higher mortality than the general population, mainly due to non-AIDS causes [6, 7]. The CD4:CD8 ratio and CD8^+^ T-lymphocyte (CD8) counts have been suggested as prognostic markers for mortality, in addition to VL and CD4 count, in such patients [8–12]. The Insight Strategic Timing of Antiretroviral Treatment (START) study group showed that in PLWH diagnosed with a CD4 count >500 cells/μL, those with a VL >50000 copies/mL or with a CD4:CD8 ratio <0.5 benefited the most from early treatment compared with deferring treatment to when CD4 count reached 350 cells/μL [13]. Moreover, they suggested that the best surrogate markers for the effect of early treatment on clinical outcomes were controlling the VL and increasing the CD4:CD8 ratio.

CD8 counts increase in response to acute infection, but also remain raised in chronic infections such as HIV. CD8 counts respond to ART more slowly than CD4 counts. In the general population, CD4:CD8 ratio declines with age and is associated with mortality [14]. The ratio is considered to be a marker of cumulative inflammation and immunological changes associated with aging [15]. Results of previous studies that examined associations of CD4:CD8 ratio or CD8 count with mortality in HIV-infected patients on ART were inconsistent [16–21].

We investigated whether the CD4:CD8 ratio or CD8 counts were independently associated with all-cause, AIDS, and non-AIDS mortality in patients treated with ART with suppressed VL and CD4 count >350 cells/μL using data from the Antiretroviral Therapy Cohort Collaboration (ART-CC).

## METHODS

### Eligibility

We combined data from 13 European and North American cohorts (see Appendix) participating in ART-CC (www.art-cohort-collaboration.org) [22]. Participation of cohorts has been approved by their ethics committees or institutional review boards according to local regulations. Cohorts use standardized methods of data collection, and schedule follow-up visits at least every 6 months.

Eligible patients were ART naive and aged ≥16 years when they started a combination ART regimen containing at least 3 antiretroviral drugs between 1996 and 2010. Follow-up began on the date after starting ART (baseline) when the patient’s CD4 count first exceeded ≥350 cells/μL, a CD8 count measurement was recorded, and VL was lower than the limit of detection of the assay or <200 copies/μL). In a sensitivity analysis, baseline was defined as the date of the second successive CD4 count ≥350 cells/μL with viral suppression. Patients were considered lost to follow-up if there was a gap of >1 year between the date the patient was last known to be alive and the cohort-specific database close date and were censored 6 months after the date they were last known to be alive. Database close date varied among cohorts between 31 May 2012 and 31 July 2013.

### Cause of Death Information

Information on mortality was obtained either through linkage with vital statistics agencies and hospitals or through physician report and active follow-up of patients. We adapted the Cause of Death (CoDe) project protocol (www.cphiv.dk/CoDe.aspx) [23] to classify causes of death. We used a computer algorithm developed by the Mortalité 2000–2005 Study Group [24] to classify deaths where *International Classification of Diseases, Tenth Revision* (ICD-10) codes were available, which were also coded by a clinician and compared. For deaths described by free text, 2 clinicians independently classified each death. Disagreements between clinicians and/or computer-assigned codes were resolved via panel discussion, as described previously [25, 26]. If information from ICD-10 codes or free text was insufficient to determine the cause, deaths were labeled “unclassifiable” and in these analyses were combined with deaths with no information and labeled “unknown.” Deaths were coded as AIDS-related if there was an AIDS-defining condition(s) close to death and/or a low CD4 count (<100 cells/μL) prior to death and a diagnosis compatible with AIDS as cause of death. All other deaths (excluding “unknown” deaths which were not included in analyses of cause-specific mortality) were assumed to be non–AIDS related.

### Statistical Methods

Covariates included in multivariable models were chosen a priori from literature [27]. We categorized age (16–29, 30–39, 40–49, 50–59, 60–69, and ≥70 years), year of ART initiation (1996–1999, 2000–2003, 2004–2007, and 2008–2010), and baseline CD4 count (350–499, 500–749, and ≥750 cells/μL). We analyzed CD4:CD8 ratio and CD8 count both as continuous variables using spline transformations to capture nonlinear associations flexibly and in tertiles of their distribution at baseline, to compare the magnitude of associations in the same metric. The knots for the cubic splines were positioned by default using Harrell recommended percentiles [28] at 0.3, 0.53, and 1.02 for CD4:CD8 ratio and at 510, 909, and 1528 cells/μL for CD8 count. The tertile groups were 0–0.40, 0.41–0.64, and >0.64 for CD4:CD8 ratio and 0–760, 761–1138, and >1138 cells/μL for CD8.

We plotted smoothed (using a moving average over 3 years) mean CD4 count, CD4:CD8 ratio, and CD8 count by time since baseline. We used Cox models stratified by cohort to estimate unadjusted, adjusted (for sex, age, people who inject drugs transmission group, calendar period of ART initiation, AIDS status, time from ART initiation to baseline) and additionally adjusted (for baseline CD4 count) mortality hazard ratios (HRs) for the lowest and highest tertiles compared to the middle (reference) tertile for baseline CD4:CD8 ratio and CD8 count. Separate models were estimated for all-cause, AIDS-related, and non-AIDS-related mortality. In the analysis of AIDS-related mortality, those experiencing non-AIDS-related death were censored at date of death (and vice versa). In a sensitivity analysis we modeled AIDS and non-AIDS mortality using competing risks regression.

We modeled and plotted adjusted HRs (compared to the median) across the range of CD4:CD8 ratios and CD8 counts using spline transformations of these variables. Spline plots were truncated at 0.1 and 1.25 for CD4:CD8 ratio (97.5% of sample) and 10 and 2000 cells/μL for CD8 count (95% of sample). We used 2 degrees of freedom likelihood ratio (LR) tests to assess the evidence that CD4:CD8 ratio and CD8 count (modeled using splines and as tertiles) were prognostic.

## RESULTS

### Characteristics

Of 66610 patients with both CD4 and CD8 counts recorded, 49865 (75%) reached a CD4 count ≥350 cells/μL with suppressed VL and therefore were included in analyses. Of these, 1834 died during 276526 person-years (6.7 deaths/1000 person-years). Two hundred forty-nine deaths (14%) were AIDS-related, 1076 (59%) non-AIDS-related, and 509 (28%) classified as unknown. Duration of ART before reaching CD4 ≥350 cells/μL was longer in those who started ART with a lower CD4 count. However, nearly half of patients in our analysis had undetectable VL and reached CD4 ≥350 cells/μL within 6 months of ART initiation (Table 1). Patient demographics and clinical characteristics at baseline are shown in Table 1. The median age was 37 (interquartile range [IQR], 31–45) years. The median baseline CD4 count, CD8 count, and CD4:CD8 ratio were 446 (IQR, 386–566) cells/μL, 930 (IQR, 679–1271) cells/μL, and 0.52 (IQR, 0.37–0.74), respectively. Baseline CD4 count was less correlated with CD4:CD8 ratio (*r* = 0.075) than with CD8 count (*r* = 0.128) (difference in correlations *P* < .0001).

**Table 1.** Characteristics (Within 3 Months) of Patients at Start of Follow-up (Baseline) (N=49865)

Variable	Patients, No. (%)
Female sex	13724 (28)
PWID transmission	3638 (7)
AIDS	9283 (19)
Age, y	
16–29	7425 (15)
30–39	18487 (37)
40–49	15204 (30)
50–59	6296 (13)
60–69	2060 (4)
≥70	393 (0.8)
Years from ART start to reaching CD4 count ≥350 cells/μL (rounded to nearest year)^a^	
0	23693 (48)
1	11440 (23)
2	4939 (10)
3	2953 (6)
4	1893 (4)
≥5	4947 (10)
Calendar period of ART initiation	
1996–1999	11739 (24)
2000–2003	11763 (24)
2004–2007	12369 (25)
2008–2012	13994 (28)
CD4 count, cells/μL	
350–499	31779 (64)
500–749	13367 (27)
≥750	4719 (9)
CD4:CD8 ratio	
0–0.40	16276 (33)
0.41–0.64	17011 (34)
>0.64	16578 (33)
CD8 count, cells/μL	
0–760	16671 (33)
761–1138	16583 (33)
>1138	16611 (33)

CD8 and CD4:CD8 have been split into tertiles calculated when the patients reach baseline. Baseline was defined as date of reaching a CD4 count ≥350 cells/μL with an undetectable viral load (lower than lower limit of viral assay or <200 copies/mL).

Abbreviations: ART, antiretroviral therapy; PWID, people who inject drugs.

^a^Year 0 contains those who reached a CD4 count ≥350 cells/μL ≤6 months after ART start.

### Trajectories of Biomarkers Over Time

**Figure 1.**
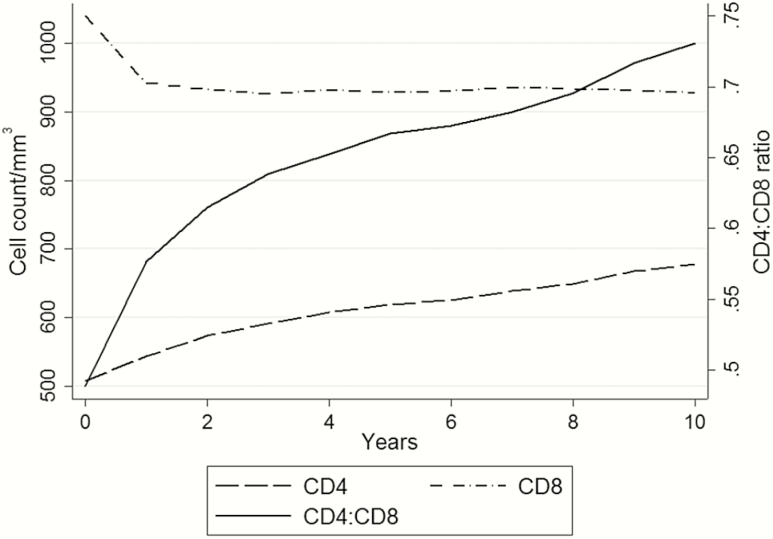
Smoothed mean CD4 count, CD8 count, and CD4:CD8 ratio, by years after baseline (reaching a CD4 count ≥350 cells/μL and viral suppression).

Smoothed mean CD4 count, CD8 count, and CD4:CD8 ratio up to 10 years after baseline are shown in Figure 1. Mean CD4 count increased steadily, from 507 to 678 cells/μL at 10 years. Mean CD8 count declined from 1040 at baseline to 942 cells/μL at 1 year, then plateaued at around 930 cells/μL. Mean CD4:CD8 ratio increased sharply from 0.49 at baseline to 0.58 at 1 year, then less rapidly to 0.73 at 10 years.

### All-Cause Mortality

**Figure 2.**
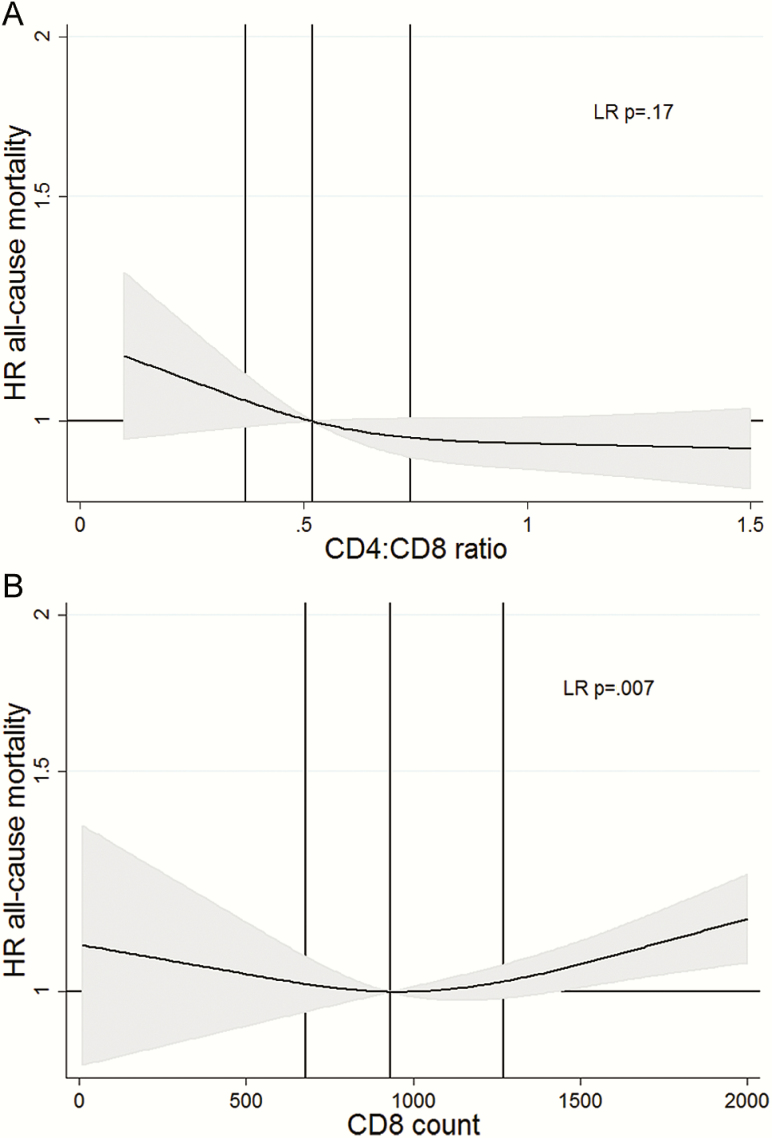
Adjusted hazard ratios (HRs) for all-cause mortality with median as comparator for CD4:CD8 ratio (upper panel) and CD8 count (lower panel), based on cubic spline models and adjusted for other prognostic variables. Gray-shaded area indicates the 95% confidence interval and the vertical lines indicate the median and interquartile range. Abbreviation: LR, likelihood ratio.

Rates of all-cause mortality declined with increasing CD4:CD8 ratio (Figure 2), but confidence intervals (CIs) were wide and there was little evidence (LR *P* = .17) that CD4:CD8 ratio was prognostic after adjusting for other variables. In contrast, CD8 count showed a U-shaped association with all-cause mortality after adjusting for other variables (LR *P* = .007), with lowest mortality rates near the median value and highest rates for those with high CD8 counts (Figure 2). Before adjustment, lower tertile of baseline CD4:CD8 ratio (hazard ratio [HR], 1.20 [95% CI, 1.08–1.34]) and higher tertile of CD8 count (HR, 1.26 [95% CI, 1.13–1.41]) were each associated with higher all-cause mortality compared with the middle (reference) tertile (Table 2), but associations were attenuated after adjustment to 1.11 (95% CI, 1.00–1.25) and 1.13 (95% CI, 1.01–1.26), respectively. Neither tertiles of CD4:CD8 ratio nor of CD8 count were prognostic for all-cause mortality (LR *P* = .16 and *P* = .11, respectively).

**Table 2.** Mortality Hazard Ratios for All-Cause, AIDS-Related, and Non-AIDS-Related Deaths Across Tertiles of CD4:CD8 Ratio and CD8 Count (N = 49865)

Mortality	CD4:CD8 Ratio			LR *P* Value	CD8 Count, Cells/μL			LR *P* Value
	0–0.40	0.41–0.64	>0.64		0–760	761–1138	>1138	
All-cause mortality								
Unadjusted	1.20 (1.08–1.34)	1	1.05 (.93–1.18)	.003	1.04 (.92–1.17)	1	1.26 (1.13–1.41)	<.001
Adjusted, no CD4^a^	1.10 (.98–1.23)	1	1.12 (1.00–1.27)	.11	1.04 (.92–1.17)	1	1.14 (1.02–1.27)	.06
Fully adjusted^b^	1.11 (1.00–1.25)	1	1.07 (.95–1.21)	.16	1.05 (.93–1.18)	1	1.13 (1.01–1.26)	.11
AIDS mortality								
Unadjusted	1.39 (1.04–1.86)	1	0.82 (.59–1.15)	.003	0.94 (.66–1.32)	1	1.55 (1.15–2.09)	.001
Adjusted, no CD4^a^	1.23 (.92–1.65)	1	0.87 (.62–1.22)	.095	0.93 (.66–1.32)	1	1.38 (1.02–1.86)	.024
Fully adjusted^b^	1.28 (.95–1.73)	1	0.77 (.54–1.10)	.016	0.94 (.67–1.33)	1	1.36 (1.01–1.84)	.037
Non-AIDS mortality								
Unadjusted	1.20 (1.04–1.39)	1	1.15 (.99–1.34)	.039	1.08 (.92–1.25)	1	1.23 (1.06–1.42)	.016
Adjusted, no CD4^a^	1.10 (.95–1.27)	1	1.24 (1.06–1.44)	.026	1.08 (.93–1.26)	1	1.11 (.96–1.29)	.35
Fully adjusted^b^	1.10 (.95–1.28)	1	1.19 (1.01–1.39)	.097	1.09 (.93–1.27)	1	1.10 (.95–1.27)	.38

Data are presented as hazard ratio (95% confidence interval) unless otherwise indicated. Deaths: all-cause = 1834; AIDS-related = 249; non-AIDS-related = 1076. Likelihood ratio *P* value is a test with 2 degrees of freedom for models containing CD4:CD8 ratio or CD8 ratio term against models without these terms.

Abbreviation: LR, likelihood ratio.

^a^The same as the fully adjusted analysis but without adjustment for CD4.

^b^Adjusted for sex, AIDS status, CD4 count, age, and viral load at baseline; time from antiretroviral therapy (ART) initiation to baseline; calendar year of ART initiation; injection drug use transmission; and stratified by cohort.

### AIDS-Related Mortality

**Figure 3.**
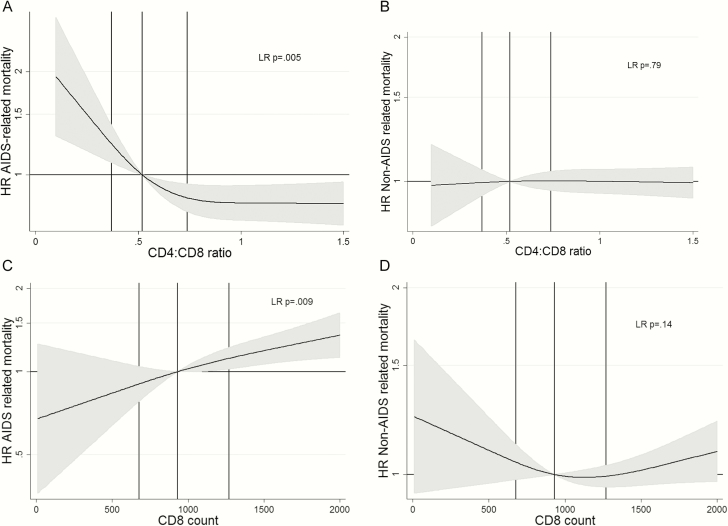
Plots of adjusted hazard ratio (HR) of AIDS-related (left panels) and non-AIDS-related (right panels) mortality with median as comparator for CD4:CD8 ratio (upper panels) and CD8 count (lower panels) with 95% confidence intervals. Modeled using cubic splines. The vertical lines indicate the median and interquartile range. Abbreviation: LR, likelihood ratio test.

AIDS-related mortality declined with increasing CD4:CD8 ratio (LR *P* = .005) and decreasing CD8 count (LR *P* = .009) (Figure 3). The adjusted hazard ratio (aHR) for AIDS-related mortality for the lowest (vs middle) tertile of CD4:CD8 ratio was 1.28 (95% CI, .95–1.73) and for the highest (vs middle) tertile of CD8 was 1.36 (95% CI, 1.01–1.84) (Table 2). Tertiles of both CD4:CD8 ratio and CD8 count were prognostic for AIDS-related deaths (LR *P* = .016 and *P* = .0037, respectively).

### Non-AIDS-Related Mortality

When CD4:CD8 ratio was modeled as a continuous variable, there was no evidence (LR *P* = .79) that it was prognostic for non-AIDS-related mortality (Figure 3). However, there was weak evidence (LR *P* = .1) that mortality was higher in the lower and upper tertiles compared with the middle tertile of CD4:CD8 ratio (aHR, 1.10 [95% CI, .95–1.28] and 1.19 [95% CI, 1.01–1.39], respectively). CD8 count had a u-shaped association with non-AIDS mortality (Figure 3), but there was little evidence that it was prognostic after adjusting for other variables (LR *P* = .14 for continuous and *P* = .38 for tertiles).

In sensitivity analyses (1) using competing risk regression and (2) starting follow-up from second CD4 count >350 cells/μL (Supplementary Table 1), estimates were similar to those in the main analysis.

## DISCUSSION

Based on a large dataset combined from European and North American clinical cohorts, we compared the utility of CD4:CD8 ratio and CD8 count as prognostic markers for mortality in PLWH on ART with suppressed VL and CD4 counts >350 cells/μL. Although mean CD8 counts decreased immediately after baseline, they plateaued at a level considered higher than normal in the general population. Mean CD4 count continued to increase over 10 years of follow-up. Mean CD4:CD8 ratio also increased during follow-up, mostly due to increases in CD4 rather than decreases in CD8 counts, but remained short of the mean in the general population: 1.8 [29]. Associations of CD4:CD8 ratio and CD8 count with all-cause mortality were attenuated after adjustment for other prognostic factors at baseline; there was little evidence that CD4:CD8 ratio was prognostic independent of CD4 count. The association of CD8 count with all-cause mortality appeared U-shaped, with higher mortality for those with higher and lower values compared with those with values near the median. AIDS-related mortality declined with increasing CD4:CD8 ratio and decreasing CD8 count. There was little evidence that CD4:CD8 ratio or CD8 count was independently prognostic for non-AIDS mortality.

### Immune Dysregulation and Inflammatory Processes

Viral infections such as HIV cause a high turnover of T cells and an accumulation of CD8 [15]. Elevated CD8 count is a marker of immune dysregulation and ongoing inflammatory processes, which may lead to increased non-AIDS-related morbidity and mortality in those treated long term for HIV infection [20]. In the general population, CD4:CD8 ratios decrease with age; many T-cell abnormalities related to aging have been observed at younger ages in HIV-infected individuals [15]. Inflammation is associated with untreated HIV infection, but ART has differential effects on markers of inflammation with some declining more than others: Interleukin 6, C-reactive protein, cystatin C, and D-dimer remain elevated with successful ART [15, 30, 31]_._

### All-Cause Mortality

Studies that followed patients for mortality from ART start have found that the dominant prognostic marker is CD4 count and the majority of deaths are AIDS related [9]. If a patient’s CD4 count is very low at ART start, then their CD8 count will also be low. This correlation between CD4 and CD8 count at ART start in immunosuppressed patients implies that the ratio is unlikely to be a useful predictor of mortality in the first year of ART [32]. However, in the START trial, which included only patients who were not immunosuppressed, low CD4:CD8 ratio was associated with the primary endpoint, which included serious AIDS and non-AIDS morbidity as well as all-cause mortality [13].

A study from the Danish HIV cohort illustrates the difficulty of interpreting CD8 count as a prognostic marker. Mortality was predicted by low CD8 count (<500 cells/μL) at ART start, but by high CD8 count 1 year afterward (>2000 cells/μL) and 10 years afterward (>1500 cells/μL) [20]. The latter 2 results are in concordance with our findings, which applied only to those with a relatively high CD4 count. Two other studies that included patients with no CD4 count restriction from ART start found that low CD8 count predicted AIDS events or all-cause mortality [3, 19]. In contrast, a large French study did not find that CD4:CD8 ratio or CD8 count added prognostic value for all-cause mortality independent of CD4 in patients with CD4 count >200 cells/μL [21], similar to our findings. These studies used a range of cutoff values for CD4:CD8 ratio or CD8 count, and did not always control for CD4 count, making comparisons difficult.

### AIDS-Related Mortality

In contrast to our study, which found that high CD8 count was associated with AIDS-related mortality, the Danish study found that low CD8 count (<500 cells/μL) 1 year after ART start was associated with an increased risk [20]. Again the different results could be due to the difference in patient populations and sample size. Interestingly, CD8 count <400 cells/μL specifically predicted occurrence of pneumonia and death in a study based in the United States [33]. In agreement with our finding of low CD4:CD8 ratio associated with AIDS-related mortality, albeit with wide CIs due to few deaths, an Italian study also found an association of low CD4:CD8 ratio with higher risk of AIDS events and AIDS-related deaths among virally suppressed patients [18].

### Non-AIDS-Related Mortality

The CD4 count measured at ART start loses its predictive value as duration of ART increases, whereas CD4:CD8 ratio has, at least in some studies, been found to predict non-AIDS morbidity and mortality after long-term ART [34]. A small case-control study reported an association between low CD4:CD8 ratio and serious non-AIDS-related events [16, 17], but in a study that included data from 4 cohorts and 3 trials, this result failed to replicate in those with high CD4 count [16]. A larger Italian study of patients with suppressed VLs found an elevated risk of non-AIDS mortality in those with CD4:CD8 <0.3 compared with between 0.3 and 0.8 [18], whereas we found little evidence that CD4:CD8 ratio or CD8 count was independently prognostic for non-AIDS mortality. Studies have also found associations between low CD4:CD8 ratio and coronary artery disease, but were underpowered to consider mortality [35].

### Strengths and Weaknesses

Our study is observational and therefore there may be unmeasured confounding, by factors such as, for example, smoking. Although there is uncertainty about the outcome of patients lost to follow-up, we would have known if they had died, as most cohorts link to death registries. Strengths of this study include prospective study design and the large sample size and numbers of deaths which were coded by a standard procedure. Our results are likely generalizable across high-resource settings as contributing cohorts came from a wide range of countries and the sample size was much larger than any previous studies of CD8 as a prognostic marker in the treated HIV-infected population [34]. Estimating associations of CD4:CD8 ratio and CD8 count using tertiles allowed us to compare HRs based on the same metric and, together with models based on cubic splines, allowed us to examine the shape of associations across the range of each measure.

### Implications

Our data show that CD8 count recovers less well than CD4 count and remains high for a lengthy period, as has been observed in other studies [20], which may indicate ongoing immune dysregulation. This implies a need for increased monitoring and early start of treatment before the CD8 count is elevated to levels it will not recover from. The consequences of this may be seen in a longer period of follow-up than we have been able to study here as our results show little evidence that CD4:CD8 is associated with all-cause mortality in a HIV population with high CD4 counts and suppressed VLs. There is slightly more evidence of an association with CD8 count and all-cause mortality in this population but the pattern was not strong despite the large size of our dataset, indicating little usefulness as a prognostic factor for all-cause mortality. The same could be said of the association of both CD4:CD8 ratio and CD8 count with non-AIDS mortality, although it is possible that associations may only be with specific causes of morbidity and death that we were unable to look at here. Our finding that both low CD4:CD8 ratio and high CD8 count were associated with AIDS mortality in this population with high CD4 counts and suppressed VLs implies that both CD4:CD8 ratio and CD8 count could account for some excess AIDS mortality in a HIV population that was otherwise healthy.

## CONCLUSIONS

Our study of patients treated with ART who are virally suppressed and have CD4 count >350 cells/μL, which is the largest to date investigating the associations of CD4:CD8 ratio and CD8 count with cause-specific mortality, does not lend strong support to using either measurement as a prognostic marker for non-AIDS related mortality. However, the failure of many patients in this long-term treated HIV-infected population to reach the levels of CD4:CD8 ratio or CD8 count considered to be normal in the general population may indicate ongoing immune dysregulation. This may have longer-term consequences than we have been able to study here, or associations may be only with specific causes of death. Therefore, these markers might contribute to the immunological evaluation of patients in long-term follow-up. Nevertheless, our results showing poor CD4:CD8 ratio recovery support treatment earlier in the course of HIV infection to better preserve immune function in PLWH as is now recommended by treatment guidelines.

## Supplementary Data

Supplementary materials are available at *Clinical Infectious Diseases* online. Consisting of data provided by the authors to benefit the reader, the posted materials are not copyedited and are the sole responsibility of the authors, so questions or comments should be addressed to the corresponding author.

## Supplementary Material

Appendix

Web_Appendix_tables

## Notes

***Author contributions.*** A. T. did the statistical analyses and wrote the first draft of the manuscript. All authors contributed to study design, collection of data, data interpretation, and writing the manuscript, and approved the final version. A. T. had full access to the data and acts as guarantor for the article. D. C. had the original concept for the study. M. T. M. and J. A. C. S. supervised the analyses.

***Acknowledgments.*** We thank all patients, doctors, and study nurses associated with the participating cohort studies.

***Financial support.*** This work was supported by the UK Medical Research Council (MRC) (grant number MR/J002380/1) and the UK Department for International Development (DFID) under the MRC/DFID Concordat agreement and is also part of the EDCTP2 programme supported by the European Union. J. A. C. S. is funded by a National Institute for Health Research Senior Investigator award (NF-SI-0611-10168). Sources of funding of individual cohorts include the Agence Nationale de Recherche sur le SIDA et les hépatites virales (ANRS); the Institut National de la Santé et de la Recherche Médicale (INSERM); the French, Italian, and Spanish Ministries of Health; the Swiss National Science Foundation (grant number 33CS30_134277); the Ministry of Science and Innovation and the Spanish Network for AIDS Research (RIS; ISCIII-RETIC RD06/006); grant to the Stichting HIV Monitoring from the Dutch Ministry of Health, Welfare and Sport through the Centre for Infectious Disease Control of the National Institute for Public Health and the Environment; the European Commission (EuroCoord grant 260694); the British Columbia and Alberta governments; the National Institutes of Health (NIH) (University of Washington Center for AIDS Research [CFAR]) (NIH grant P30 AI027757); The University of Alabama at Birmingham CFAR (NIH grant P30-AI027767); the Tennessee CFAR (NIH grant P30 AI110527); the National Institute on Alcohol Abuse and Alcoholism (U10-AA13566, U24-AA020794); the US Department of Veterans Affairs; the Michael Smith Foundation for Health Research; the Canadian Institutes of Health Research; and the Veterans Health Administration Office of Research and Development. The Danish HIV Cohort Study is founded by the Preben and Anne Simonsens Foundation. Data from 11 European cohorts were pooled in June 2014 within COHERE in EuroCoord (www.cohere.org and www.EuroCoord.net). COHERE receives funding from the European Union Seventh Framework Programme (FP7/2007–2013) under EuroCoord grant agreement 260694.

***Potential conflicts of interest.*** This work was funded by unrestricted grants from Abbott, Gilead, Tibotec-Upjohn, ViiV Healthcare, MSD, GlaxoSmithKline, Pfizer, Bristol-Myers Squibb, Roche, and Boehringer-Ingelheim. All authors report no other potential conflicts. All authors have submitted the ICMJE Form for Disclosure of Potential Conflicts of Interest. Conflicts that the editors consider relevant to the content of the manuscript have been disclosed.

## APPENDIX

***Contributing cohorts.*** Cohorts included in this article were the French Hospital Database on HIV (FHDH); the Italian Cohort of Antiretroviral-Naive patients (ICONA); the Swiss HIV Cohort Study (SHCS); the AIDS Therapy Evaluation project, The Netherlands (ATHENA); the Aquitaine Cohort; the Royal Free Hospital Cohort; the South Alberta Clinic Cohort; ART Observational Medical Evaluation and Research (HOMER), Canada; HIV Atlanta Veterans Affairs Cohort Study (HAVACS), United States; Osterreichische HIV-Kohortenstudie (OEHIVKOS), Austria; Vanderbilt, United States; 1917 Clinic Cohort, University of Alabama, Birmingham, United States (UAB); and the Koln/Bonn Cohort.

***ART-CC steering group.*** Andrew Boulle (IeDEA Southern Africa), Christoph Stephan (Frankfurt), Jose M. Miro (PISCIS), Matthias Cavassini (SHCS), Geneviève Chêne (Aquitaine), Dominique Costagliola (FHDH), François Dabis (Aquitaine), Antonella D’Arminio Monforte (ICONA), Julia del Amo (CoRIS-MD), Ard Van Sighem (ATHENA), Jorg Janne Vehreschild (Koln/Bonn), John Gill (South Alberta Clinic), Jodie Guest (HAVACS), David Hans-Ulrich Haerry (EATG), Robert Hogg (HOMER), Amy Justice (VACS), Leah Shepherd (EuroSIDA), Niels Obel (Denmark), Heidi M. Crane (Washington), Colette Smith (Royal Free), Peter Reiss (ATHENA), Michael Saag (Alabama), Tim Sterling (Vanderbilt-Meherry), Ramon Teira (VACH), Matthew Williams (UK-CAB), Robert Zangerle (Austria).

***ART-CC coordinating team.*** Jonathan Sterne and Margaret May (principal investigators), Suzanne Ingle and Adam Trickey (statisticians).
