# Supplementary material for: CD4:CD8 Ratio and CD8 Count as Prognostic Markers for Mortality in Human Immunodeficiency Virus–Infected Patients on Antiretroviral Therapy: The Antiretroviral Therapy Cohort Collaboration (ART-CC)
Source: Clin Infect Dis. 2017 Jul 11;65(6):959–66. doi: 10.1093/cid/cix466 (PMC5850630; doi:10.1093/cid/cix466)
Supplement: Appendix [file cix466_suppl_appendix.docx]

**Appendix: Contributing cohorts**

Cohorts included in this paper were the French Hospital Database on HIV (FHDH); the Italian Cohort of Antiretroviral-naïve patients (ICONA); the Swiss HIV Cohort Study (SHCS); the AIDS Therapy Evaluation project, Netherlands (ATHENA); the Aquitaine Cohort; the Royal Free Hospital Cohort; the South Alberta Clinic Cohort; ART Observational Medical Evaluation and Research (HOMER), Canada; HIV Atlanta Veterans Affairs Cohort Study (HAVACS), US; Osterreichische HIV-Kohortenstudie (OEHIVKOS), Austria; Vanderbilt, US; 1917 Clinic Cohort, University of Alabama, Birmingham, US (UAB); and the Koln/Bonn Cohort.

**ART-CC Steering group**

Andrew Boulle (IeDEA Southern Africa), Christoph Stephan (Frankfurt), Jose M. Miro (PISCIS), Matthias Cavassini (SHCS), Geneviève Chêne (Aquitaine), Dominique Costagliola (FHDH), François Dabis (Aquitaine), Antonella D’Arminio Monforte (ICONA), Julia del Amo (CoRIS-MD), Ard Van Sighem (ATHENA), Jorg Janne Vehreschild(Koln/Bonn), John Gill (South Alberta Clinic), Jodie Guest (HAVACS), David Hans-Ulrich Haerry (EATG), Robert Hogg (HOMER), Amy Justice (VACS), Leah Shepherd (EuroSIDA), Niels Obel (Denmark), Heidi M Crane (Washington), Colette Smith (Royal Free), Peter Reiss (ATHENA), Michael Saag (Alabama), Tim Sterling (Vanderbilt-Meherry), Ramon Teira (VACH), Matthew Williams (UK-CAB), Robert Zangerle (Austria)

**ART-CC Co-ordinating team**

Jonathan Sterne and Margaret May (Principal Investigators), Suzanne Ingle, Adam Trickey (statisticians).
