# Supplementary material for: CD4:CD8 Ratio and CD8 Count as Prognostic Markers for Mortality in Human Immunodeficiency Virus–Infected Patients on Antiretroviral Therapy: The Antiretroviral Therapy Cohort Collaboration (ART-CC)
Source: Clin Infect Dis. 2017 Jul 11;65(6):959–66. doi: 10.1093/cid/cix466 (PMC5850630; doi:10.1093/cid/cix466)
Supplement: Web_Appendix_tables [file cix466_suppl_web_appendix_tables.docx]

**Web Appendix tables**

**Web table 1:** Mortality hazard ratios for all-cause, AIDS-related, and non-AIDS-related deaths across tertiles of (i) CD4:CD8 and (ii) CD8 count. Follow-up starts when patients reach their second successive CD4≥350 cells/mm^3^ and have an undetectable viral load. N=45,009 Deaths: all-cause=1,522, AIDS-related=183, non-AIDS-related=885.

|  | **Categories of CD4:CD8 ratio** | | |  | **Categories of CD8 count (cell count/mm^3^)** | | |
| --- | --- | --- | --- | --- | --- | --- | --- |
|  | 0-0.40 | 0.41-0.64 | >0.64 |  | 0-760 | 761-1138 | >1138 |
| **All-cause mortality** |  |  |  |  |  |  |  |
| Unadjusted | 1.19 (1.05, 1.35) | 1 | 1.00 (0.88, 1.13) |  | 1.02 (0.89, 1.16) | 1 | 1.26 (1.12, 1.42) |
| Adjusted, no-CD4* | 1.06 (0.93, 1.20) | 1 | 1.06 (0.94, 1.20) |  | 1.03 (0.91, 1.18) | 1 | 1.14 (1.01, 1.29) |
| Fully adjusted** | 1.08 (0.95, 1.22) | 1 | 1.01 (0.89, 1.15) |  | 1.05 (0.92, 1.19) | 1 | 1.12 (0.99, 1.27) |
| **AIDS mortality** |  |  |  |  |  |  |  |
| Unadjusted | 1.20 (0.86, 1.68) | 1 | 0.67 (0.46, 0.98) |  | 0.82 (0.55, 1.22) | 1 | 1.53 (1.09, 2.15) |
| Adjusted, no-CD4* | 1.05 (0.75, 1.48) | 1 | 0.69 (0.48, 1.01) |  | 0.84 (0.56, 1.25) | 1 | 1.35 (0.96, 1.91) |
| Fully adjusted** | 1.11 (0.78, 1.56) | 1 | 0.63 (0.42, 0.92) |  | 0.85 (0.57, 1.27) | 1 | 1.33 (0.94, 1.88) |
| **Non-AIDS mortality** |  |  |  |  |  |  |  |
| Unadjusted | 1.17 (0.99, 1.38) | 1 | 1.09 (0.93, 1.28) |  | 1.04 (0.88, 1.23) | 1 | 1.24 (1.06, 1.45) |
| Adjusted, no-CD4* | 1.06 (0.90, 1.25) | 1 | 1.16 (0.99, 1.36) |  | 1.06 (0.90, 1.26) | 1 | 1.12 (0.96, 1.32) |
| Fully adjusted** | 1.06 (0.90, 1.26) | 1 | 1.11 (0.94, 1.31) |  | 1.07 (0.91, 1.27) | 1 | 1.11 (0.95, 1.31) |

*The same as the fully adjusted analysis** but without adjustment for CD4

**adjusted for gender, AIDS status, CD4 count, age, and viral load at baseline, time from ART initiation to baseline, calendar year of ART initiation, injecting drug use transmission, and stratified by cohort.
